# Canalis Sinuosus Damage after Immediate Dental Implant Placement in the Esthetic Zone

**DOI:** 10.1155/2019/3462794

**Published:** 2019-12-16

**Authors:** Roman Volberg, Oleg Mordanov

**Affiliations:** ^1^Private Practice, Moscow, Russia; ^2^Department of Prosthodontics, Central Research Institute of Dental and Maxillofacial Surgery, Moscow, Russia

## Abstract

Dental implant failure in the anterior maxilla can be caused by the range of the features. One of them is neighboring neurovascular structure damage, such as the canalis sinuosus (CS), that carries the superior anterior alveolar nerve. The aim of the report is to demonstrate clinical symptomatology and radiographic signs of CS damage in a 45-year-old female patient who underwent upper left lateral incisor extraction and immediate implant placement and implant removal in 16 days secondary to pain and paresthesia in the maxillary left region.

## 1. Introduction

Immediate implant placement has been considered as an effective treatment for partially edentulous patients with a high implant survival rate [1]. However, not all implants that survive are necessarily successful [2]. Implant failure may be primary or secondary nature [3]. One of the examples of the secondary implant failure and, as a result, untoward consequence of performing implant dentistry could be the implant removal due to the damage to the infraorbital nerve and its branches [4].

The anterior superior alveolar nerve (ASAN) is a branch of infraorbital nerve that enters in the infraorbital canal, which has a side intrabony branch called the *canalis sinuosus* (CS) [5, 6]. The canalis sinuosus goes through the anterior wall of the maxilla and then along the lateral wall of the nasal cavity [7], residing in the alveolar process of the maxilla [8–12]. CS nerves and vessels supply anterior teeth and adjacent soft tissues [13].

The proximity to the neurovascular bundle of the CS can compromise dental implant treatment [8, 14, 15] with potential bleeding and temporary or permanent sensory disturbances [9, 16]. For example, Machado et al. [9] presented two case reports where patients suffered from pain and it was immediately relieved after implant extractions that had been placed with CS damage. Also, McCrea [14] and Arruda et al. [17] reported different cases in which dental implant placement in the anterior maxilla in the CS led to persistent pain, and the patient's symptoms were resolved with the following removal of the implant. However, Shaeffer [18] reported a case of intractable pain following implant placement in the upper left first premolar region and the pain did not subside following the extraction of the implant.

When planning dental implants, carrying out radiographic examinations, alongside clinical examinations, has become necessary to reduce the risk of implant procedure failure and complications. The cone-beam computed tomography (CBCT) imaging is a valuable tool to determine the anatomic structures before any surgery, including implant surgery [19]. The recent literature review stated that CBCT exams were the best way to evaluate the CS [16].

Also, it was shown that the terminal portion of CS is more prevalent in the anterior region of the maxilla, more specifically in the incisor and canine regions near the palate [16]. On the other hand, the widely recommended direction for immediate implant placement in the anterior maxilla is palatal to the extracted root axis to engage more native bone in order to achieve maximum bony support and favorable esthetic outcome [20].

The aim of this article is to report a case with clinical and radiographic signs of CS damage after immediate dental implant placement in the maxillary left lateral incisor region.

## 2. Case Report

A 45-year-old female patient underwent maxillary left lateral incisor extraction secondary to severe horizontal tooth fracture below the cementoenamel junction (CEJ). Preoperative CBCT examination was provided and evaluated before the procedure.

The CBCT scan was obtained with a 3D eXam (KaVo, Biberach, Germany) with standard exposure settings (23 cm × 17 cm field of view, 0.3 mm voxel size, 110 kV, and 1.6–20 s) and was analyzed with the I-CAT viewer software (Version 10, Hatfield, England).

**Figure 1:**
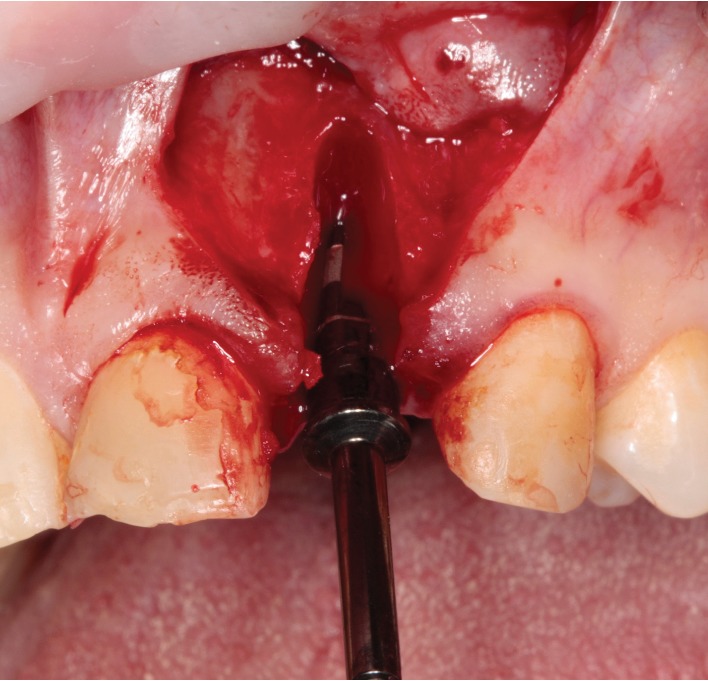
Intraoperative view. Implant site preparation after upper left lateral incisor extraction. No excessive bleeding was presented during the surgery.

**Figure 2:**
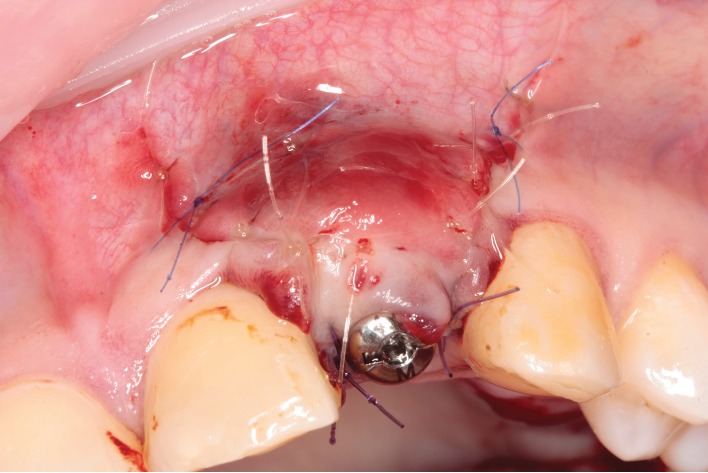
Intraoperative view. Immediate implant placement in the upper left lateral incisor region after GBR and soft tissue grafting. The healing abutment was placed and flaps were sutured.

The patient gave written consent before all procedures. Extraction was provided atraumatically with the luxators. The flap was reflected. Immediate implant placement was performed with the simultaneous guided bone regeneration (GBR) without corticotomy and soft tissue grafting (so-called “Burger technique” by Urban et al. [21, 22]). The implant placement and GBR procedures went without any complications (Figure 1); primary stability of the implant was 35 N cm (Dentium Implantium 3.5 mm∗10 mm). The GBR was provided using a mixture of the autogenous bone and a Bio-Oss® particulate graft (Geistlich) and Bio-Gide membrane (Geistlich). The site was sutured with Prolene 6/0. No excessive bleeding, as in the case of vessel damage, was noted during the surgery (Figure 2).

In a few hours after the procedure, the patient started complaining about the pain and paresthesia in the left maxilla. First week pain was insignificant, and 400 mg of ibuprofen was prescribed twice a day and it relieved the pain. In one week, the pain started increasing and became so strong and continuous that none of the painkillers could relieve it.

The pain generally was located in the area of the maxillary left canine. All sensitive disorders were not located in the area of implant site—they spread out around the maxillary left canine, the palate, and the nasal region. Also, the patient complained about burning in the back of the head in the occipital region. Painkillers, tranquilizers, and neurotropic agents were ineffective at this moment. However, no significant findings were noted during extraoral and intraoral clinical examinations.

After the clinical examinations, it was suspected to be a neurological disorder, so differential diagnostic procedures were held. During these procedures, different types of the nerve blocks were performed on the patient. Palatal, incisive, and infraorbital nerve blocks were used one by one; however, only the infraorbital block relieved the pain.

No other symptoms of inflammation were found such as pain, fever, exudation, or excessive bleeding in the area of implant placement and GBR. Neither the patient nor her relatives presented neurological disease in the medical history.

**Figure 3:**
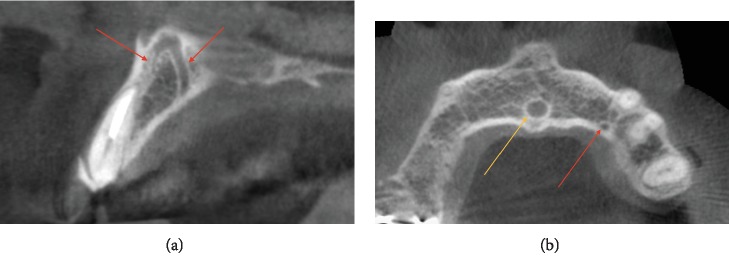
Preoperative CBCT scan. (a) Sagittal view. Accessory branches of CS (red arrows) have palatal and buccal directions close to the maxillary left lateral incisor. (b) Axial view. CS (red arrow) near the maxillary left canine and nasopalatine canal (yellow arrow).

**Figure 4:**
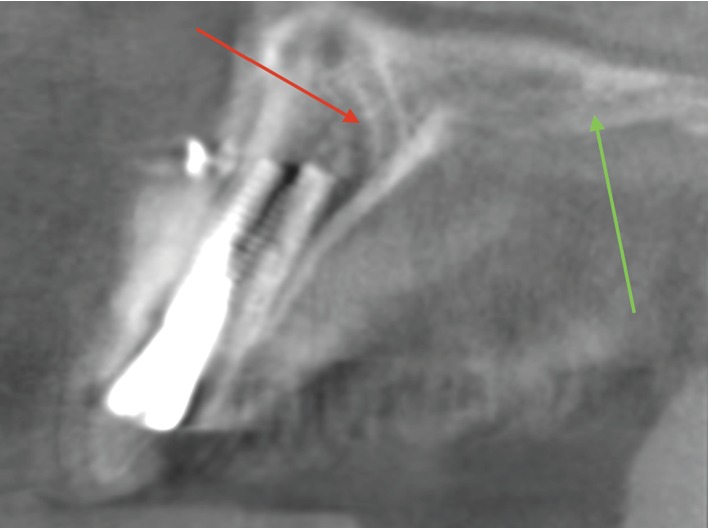
Postoperative CBCT scan, sagittal view in the left maxillary incisor region. The dental implant in situ. The diagnosis is difficult due to artefacts caused by the titanium implant; however, the major palatal branch of the CS could be seen (red arrow). Also, the bony contours of the left greater palatal artery and nerve are visualized (green arrow).

New CBCT evaluation was performed after the surgery with the same equipment. During precise examination of the CBCT scans before and after the implant placement, CS was found with the diameter 2.3 mm in size with two small branches. One of them was found close to the palatal side with the diameter 1.9 mm in size; the other was found close to the buccal side in the maxillary left lateral incisor/implant region (Figures 3 and 4) with the diameter 0.7 mm in size in the most coronal point (both were referred to the lateral incisor region according to the de Oliveira-Santos et al. classification [5]).

**Figure 5:**
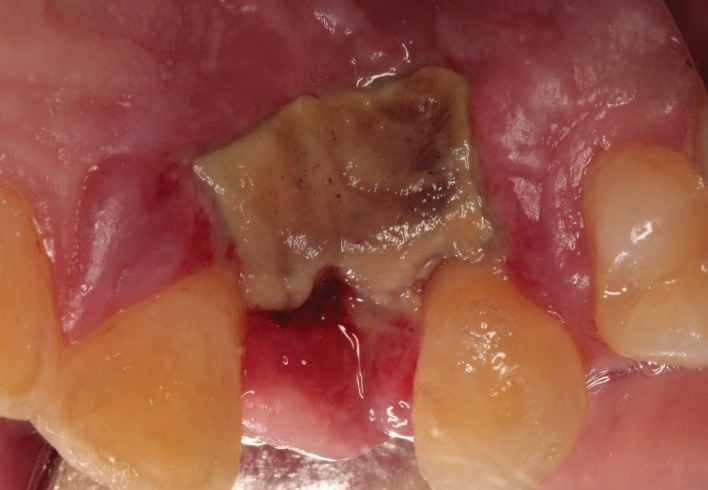
Palatal mucosa necrosis after dental implant extraction.

The implant was extracted in two weeks and two days. Then, the pain and paresthesia started slowing down. However, an area of necrosis was found by the end of the third week on the palatal mucosa (Figure 5) even though the palatal flap was not reflected and no palatal and nasopalatine anesthesia was provided, so palatal blood supply was not compromised. Also, bony contours of the left greater palatal artery and nerve were visualized on the CBCT scans in the anterior maxilla 10.1 mm from the implant (Figure 5); thus, the damage of the greater palatal artery was excluded. The implant was 5.62 mm from the incisive foramen and 5.66 mm from the nasopalatine canal according to CBCT data, so the intervention to these structures is excluded as well.

The bony defect after implant removal healed under a blood clot as an alveolar socket after tooth extraction. The patient was prescribed antiseptics for local application. Further treatment and follow-ups of the patient were impossible, because the patient decided to change the clinical practice.

## 3. Discussion

The CS, first described by Jones [23], carries an anterior superior neurovascular bundle through the anterior maxilla that innervates the central and lateral incisors and the canines. This small, poorly recognized bony canal is not known by surgeons unless complications such as paresthesia happen [11].

Several studies and clinical cases showed that CBCT is the best radiographic technique for CS visualization [16]. The CBCT examinations in the case showed that the accessory damaged canal of canalis sinuosus was buccal to the lateral incisor; according to von Arx et al. [12] and Machado et al. [9], the end of the CS was most frequently found palatal to the anterior maxillary teeth especially to the central incisors.

Though CBCT provides accurate three-dimensional images of dentomaxillofacial hard tissues [24], its limitation is the presence of image artefacts—deviations between the reconstructed image and the real content of the studied object [25]. A dental implant is a high-density structure that is the source of beam hardening resulting in the artefacts [26]. That is why after suspecting CS damage in the postoperative CBCT scans, it was decided to evaluate the preoperative CBCT scan for better diagnosis without the implant artefact.

During the preoperative CBCT evaluation and dental implant planning, none of the nerve structures around left lateral incisor were noticed. This demonstrates the necessity of knowing the existence of CS and its characteristics, which may influence treatment outcomes.

There are several case reports with dental implants placed in the anterior maxilla and CS damage, presenting the same neurological symptomatology [9, 17]; however, this case additionally contributes the symptoms of trigeminal neuralgia [27] as pain irradiation in the back of the head and no intraoperative excessive bleeding rather than the usual one during the implant site preparation. The tests with the different types of local nerve blocks showed a good diagnostic efficacy indicating the damaged infraorbital nerve branch.

As the damage of the greater palatal and nasopalatine nerve and blood supply was excluded, the palatal mucosa necrosis that appeared after implant extraction could be explained by the contraction of smooth muscle within the arterial wall during the neurovascular bundle constriction that leads to transient ischemia of structures and tissue necrosis [28]. However, the definite terms of the necrosis manifestation are still unclear.

To avoid sensory disturbance in the anterior maxilla in the case of CS detection, Shelley et al. [29] in their case offered a realistic fixed alternative: an adhesive cantilever bridge that had the advantage of very low risk, rapidity of production, and low cost, to potentially damaging implant therapy.

## 4. Conclusion

This clinical report of immediate implant placement showed the importance of anatomical structure knowledge and accurate and precise preoperative CBCT scan estimation, especially in the esthetic zone. The damage of CS can lead to neurological symptomatology and implant extraction.

We suggest that single-tooth defects with the risk of CS damage in the region should be restored without implant treatment or with the use of surgical guides designed for fully guided implant placement.
